# Phosphorus Restriction Changes the Expression of Fibroblast Growth Factor 23 and Its Receptors in Laying Hens

**DOI:** 10.3389/fphys.2020.00085

**Published:** 2020-02-14

**Authors:** Zhouzheng Ren, Jiakun Yan, Qianli Hu, Xinshuai Liu, Chong Pan, Yanli Liu, Xiaozhen Zhang, Xin Yang, Xiaojun Yang

**Affiliations:** College of Animal Science and Technology, Northwest A&F University, Yangling, China

**Keywords:** dietary phosphorus, fibroblast growth factor 23, fibroblast growth factor receptor, klotho, laying hen

## Abstract

Dietary phosphorus oversupply wastes non-renewable natural resources and raises environmental concerns in animal agriculture. We hypothesized that laying hens do not need large safety margins for dietary phosphorus because of the existence of fibroblast growth factor 23 (FGF23). In experiment 1, a total of 504 Hy-Line Brown laying hens (40-week-old) were randomly assigned to seven diets (for each diet, six replicates of 12 hens), containing 0.12, 0.17, 0.22, 0.27, 0.32, 0.37, and 0.42% non-phytate phosphorus, respectively, for 15 weeks. In experiment 2, a total of 14 Hy-Line Brown laying hens (40-week-old) were randomly assigned to two diets: (1) phosphorus restricted (*n* = 7) diet containing 0.14% non-phytate phosphorus, and (2) regular phosphorus (*n* = 7) diet containing 0.32% non-phytate phosphorus, for 21 days. Laying performance and egg quality were investigated in experiments 1 and 2. Phosphorus excretion and physiological changes were determined in experiment 2. It was found that dietary non-phytate phosphorus levels had no effects (*P* > 0.05) on laying performance and egg quality in either experiment. In experiment 2, laying hens fed 0.14% non-phytate phosphorus had decreased phosphorus excretion (by 52.6%, *P* < 0.001) when compared to those fed 0.32% non-phytate phosphorus. In response to the 0.14% non-phytate phosphorus diet, laying hens in experiment 2 exhibited: (1) suppressed calvaria mRNA expressions of *FGF23* (by 57.8%, *P* < 0.001) and fibroblast growth factor receptor 1 (*FGFR1*, by 52.8%, *P* = 0.012), (2) decreased serum levels of FGF23 (by 41.7%, *P* = 0.011) and phosphorus (by 40.3%, *P* < 0.001), (3) decreased kidney mRNA expressions of *FGFR1* (by 66.0%, *P* = 0.040) and *FGFR4* (by 63.3%, *P* = 0.012) and decreased kidney protein expression of type 2a sodium-phosphorus co-transporter (NPt2a, by 51%, *P* = 0.025), (4) increased duodenum protein expression of NPt2b (by 45%, *P* = 0.032), and (5) increased excretion of calcium (by 22.9%, *P* ≤ 0.024). Collectively, decreasing dietary non-phytate phosphorus by up to 0.12% had no negative effects on egg-production performance but significantly decreased phosphorus excretion in laying hens. The laying hens adjusted to low-phosphorus diets by increasing intestinal NPt2b protein production, which was associated with decreased serum FGF23 concentration. Decreasing dietary non-phytate phosphorus is suggested to laying-hen nutritionists.

## Introduction

Phosphorus homeostasis and excretion are of particular interest in agricultural animals (Fink et al., 2018; Goyette et al., 2018). The phosphorus homeostasis system allows animals to adjust to a wide range of dietary phosphorus consumption levels (Jing et al., 2018). In animal agriculture, in order to avoid the risk of deficiency symptoms, dietary phosphorus input level is generally set with a safety margin far above the ordinary requirement (Bougouin et al., 2014). As a result, phosphate rock, a non-renewable resource that is depleting rapidly (Vaccari et al., 2019), is supplemented into animal feed, and runoff from animal wastes causes environmental issues like algal blooms in the surface waters of the watersheds of intensively farmed animals (Martin et al., 2018; Mekonnen and Hoekstra, 2018). In a recent study, researchers have shown that a reduction in dietary non-phytate phosphorus level, from 0.45 (industry level in Canada) to 0.15%, had no adverse effects on health and egg-production performance but significantly decreased phosphorus excretion in egg-laying hens (Jing et al., 2018). Similarly, in a previous study (Cheng et al., unpublished results), we found that 0.14% non-phytate phosphorus is adequate for egg-laying hens from 29 to 40 weeks of age. Obviously, the adaptability of egg-laying hens to low-phosphorus diets has been largely underestimated by poultry nutritionists. Investigating the homeostatic molecular responses to low-phosphorus diets would help us to understand why decreasing dietary phosphorus input is a physiologically reasonable, cost-effective, and environmentally friendly nutrition strategy for producing laying-hen diets.

Body phosphorus homeostasis is delicately controlled by complex interactions among different organs/tissues, including but not limited to intestine, kidney, bone, and parathyroid gland (Michigami et al., 2018). Historically, decades of research have been devoted to studying the systemic importance of PTH and 1,25(OH)_2_D_3_ in phosphorus-calcium homeostasis (Portale et al., 1984). In recent years, efforts to conquer phosphorus absorption/excretion disorders in humans have led to the discoveries of several novel peptides, so-called phosphatonins (Edmonston and Wolf, 2019), which are directly involved in phosphorus homeostasis. Of particular note is the discovery of FGF23 (White et al., 2000), a bone-derived phosphatonin that plays a central role in the regulation of phosphorus homeostasis by inhibiting the expression levels of intestinal type 2b sodium-phosphorus co-transporter (NPt2b, responsible for intestinal phosphorus absorption) (Bai et al., 2003) and renal NPt2a and NPt2c (responsible for renal phosphorus resorption) (Gattineni et al., 2009). The secretion of FGF23 is very sensitive to and positively correlated to dietary phosphorus levels (Perwad et al., 2005). We have previously shown that FGF23 creates a plasma phosphate ceiling in laying hens (Ren et al., 2017b). Increasing dietary phosphorus levels stimulates the secretion of FGF23, which subsequently triggers phosphorus excretion to avoid phosphorus toxicity (Ren et al., 2017a). Conversely, at lower dietary phosphorus levels, the body suppresses the secretion of FGF23 and subsequently enhances phosphorus retention to avoid phosphorus deficiency (Levi et al., 2019). In this sense, the existence of FGF23 explains very well why over-supplementation of phosphorus in animal diets is nutritionally meaningless and indeed increases the burden on the body and the natural environment.

Fibroblast growth factor 23 acts through the klotho- FGFR complex (Michigami et al., 2018). Klotho serves as a co-receptor and converts the classic FGFR into FGF23-specific receptors (Urakawa et al., 2006). It is reported that FGF23 could activate all of the four FGFR types (FGFR1-4) with the presence of klotho protein (Kuro-o, 2019). In the current study, the responses of FGF23 and its receptors to dietary phosphorus variations were examined in laying hens. We hypothesized that laying hens can adapt well to a low-phosphorus corn-soybean meal-based diet (with no extra inorganic phosphorus supplementation) by adjusting circulating levels of FGF23. Our objectives were to (1) determine the effects of dietary non-phytate phosphorus levels on egg-production performance and phosphorus excretion in laying hens, and (2) reveal the physiological mechanisms related to the adaptability of laying hens to low-phosphorus diets.

## Experimental Methods

### Animal Care

All experimental procedures involving animals were approved by the College of Animal Science and Technology Animal Care and Use Committee at Northwest A&F University (ethical approval number NWAFAC1008) and were performed in accordance with the guidelines. The birds were individually housed in laying-hen cages with raised wire floors (depth × width × height = 45 cm × 35 cm × 45 cm) in the animal nutrition research laboratory at the College of Animal Science and Technology, Northwest A&F University. Feed and fresh water were supplied *ad libitum*. Sixteen hours of lighting (05:30 am to 09:30 pm; a combination of natural and artificial lighting was used) was provided every day. Egg production, egg weight, and feed intake were recorded daily. Laying rate, average egg weight, average feed intake, and feed-to-egg ratio were calculated accordingly. On the last day of both experiments 1 and 2, eggs were collected for the determination of egg quality parameters using previously described techniques (Ren et al., 2016, 2018, 2019).

### Experiment 1

Experiment 1 was conducted to demonstrate the adaptability of laying hens to low-phosphorus diets. Hy-Line Brown laying hens (40 weeks old, *n* = 504) were randomly allotted into seven treatments with six replicates of 12 birds. Seven experimental diets were prepared using a corn-soybean meal-based laying-hen diet (Table 1, with no inorganic phosphorus supplementation) containing 0.34% total phosphorus and 0.12% non-phytate phosphorus. Dicalcium phosphate was added into the basal diet to provide 0, 0.05, 0.10, 0.15, 0.20, 0.25, and 0.30% non-phytate phosphorus, making the final non-phytate phosphorus levels of the experimental diets to 0.12, 0.17, 0.22, 0.27, 0.32, 0.37, and 0.42%, respectively. The levels of calcium carbonate and sand were adjusted to keep dietary calcium levels consistent among treatments. The feeding trial lasted for 15 weeks. Laying performance (measured as laying rate, egg weight, daily feed intake, feed-to-egg ratio, and unqualified egg rate) and egg quality (measured as eggshell thickness, eggshell strength, and egg shape index) parameters were determined.

**TABLE 1:** Composition and nutrient levels of basal diets used in experiments 1 and 2.

Items (%, unless noted)	Experiment 1	Experiment 2
**Ingredient**		
Corn	60.53	59.76
Soybean meal	15.09	23.00
Corn germ meal	4.00	–
Corn gluten meal	3.00	–
Distillers dried grains with solubles	4.00	4.00
Calcium carbonate	9.60	9.22
Dicalcium phosphate	–	–
Soybean oil	0.47	1.25
Sodium chloride	0.20	0.25
L-Lysine-H_2_SO_4_	0.26	0.13
L-Threonine	–	0.31
DL-Methionine	0.16	0.25
Tryptophan	0.08	–
Choline chloride	0.08	0.10
Sand	2.00	0.73
Premix*	0.53	1.00
In total		100.00
**Nutrient levels**		
Metabolizable energy (calculated, kcal/kg)	2650	2700
Crude protein (calculated/analyzed)	15.8/16.0	16.5/16.9
Total phosphorus (calculated/analyzed)	0.34/0.32	0.33/0.30
Non-phytate phosphorus (calculated)	0.12	0.14
Calcium (calculated/analyzed)	3.80/3.69	3.50/3.45

*Provided per kg of diet. Experiment 1: iron 54 mg, manganese 70.5 mg, copper 8.4 mg, zinc 49.5 mg, vitamin A 8050 IU, vitamin D_3_ 2415 IU, vitamin E 24.2 mg, vitamin K_3_ 2.0 mg, thiamine 1.7 mg, riboflavin 5.2 mg, nicotinamide 10.6 mg, and pyridoxine 3.7 mg. Experiment 2: manganese 60 mg, copper 8 mg, zinc 80 mg, iodine 0.35 mg, selenium 0.3 mg, vitamin A 8000 IU, vitamin D_3_ 1600 IU, vitamin E 30 mg, vitamin K_3_ 1.5 mg, thiamine 4 mg, riboflavin 13 mg, pantothenic acid 15 mg, nicotinamide 20 mg, pyridoxine 6 mg, biotin 0.15 mg, folic acid 1.5 mg, and cobalamin 0.02 mg.

### Experiment 2

#### Experimental Procedures

Hy-Line Brown layers (40 weeks old, *n* = 14, with an average body weight of 1870 g) were randomly allotted to one of two dietary treatments: (1) restricted dietary phosphorus (*n* = 7), where laying hens were fed with a corn-soybean meal-based laying-hen diet with no inorganic phosphorus supplementation (Table 1) and total dietary phosphorus and non-phytate phosphorus levels were 0.33 and 0.14%, respectively; (2) regular dietary phosphorus (*n* = 7), where dicalcium phosphate was added into the basal diet to provide 0.20% non-phytate phosphorus, and total dietary phosphorus and non-phytate phosphorus levels were 0.51 and 0.32%, respectively. It is speculated in the Chinese Feeding Standard of Chicken (NY/T 33-2004) that the dietary non-phytate phosphorus requirement of laying hens is around 0.32%. The levels of calcium carbonate and sand were adjusted to keep dietary calcium level consistent within treatments. The feeding trial lasted for 21 days. At the end of the trial: (1) 24-h total excreta from each laying hen was collected for the determination of phosphorus and calcium excretion; (2) the laying hens were bled for serum samples and humanely euthanized for calvaria, liver, intestinal mucosa (duodenum, jejunum, and ileum), and kidney. The samples were collected immediately after euthanasia of each laying hen, frozen in liquid nitrogen, and then transferred to a −77°C freezer until further analysis (quantitative real-time PCR and/or Western blot).

#### Serum Analysis

At the end of the trial, blood samples (5 mL) were collected from the wing vein using vacuum tubes without anticoagulant. Blood samples were clotted for 20 min at room temperature and then centrifuged (594 *g*) for 15 min at 4°C. Serum samples were separated, aliquoted into Eppendorf tubes (300 μL each), and stored at −80°C. For the determination of serum phosphorus concentration, samples were mixed with molybdic acid to generate phosphomolybdic acid, which was then restored to molybdenum blue for colorimetric analysis, following the manual provided by the kit supplier (catalog no. C006-3, Nanjing Jiancheng Bioengineering Institute, Nanjing, China). For the determination of serum calcium concentration, samples were reacted with Methyl Thymol Blue using a commercial colorimetric kit (catalog no. C004-2) purchased from Nanjing Jiancheng Bioengineering Institute. Serum AKP activity was analyzed colorimetrically using a kit (catalog no. A059-2) purchased from Nanjing Jiancheng Bioengineering Institute. Briefly, AKP decomposes disodium phenyl phosphate to produce phenol, which reacts with 4-aminoantipyrine and is oxidized to a red complex of quinone derivative. One unit of AKP (King unit) represents the amount of AKP that reacts with the matrix to produce 1 mg phenol in 15 min at 37°C. Serum concentrations of FGF23 (catalog no. ml00321112), 1,25(OH)_2_D_3_ (catalog no. ml00697414), and parathyroid hormone (PTH, catalog no. ml00987411) were determined by sandwich enzyme-linked immunosorbent assays using commercial kits purchased from Meilian Biological Technology Co., Ltd. (Shanghai, China) following the manufacturer’s instructions. Spectrophotometric analysis was accomplished using either a Synergy HT plate reader (BioTek, Winooski, VT; for the determination of serum calcium, AKP, FGF23, 1,25(OH)_2_D_3_, and PTH levels) or a UV-1800 spectrophotometer (Shimadzu, Japan; for the determination of serum phosphorus levels).

#### Phosphorus and Calcium Excretion

Excreta samples were oven-dried, air equilibrated, weighed, and ground. The phosphorus content of the excreta samples was determined colorimetrically with ammonium-vanadium-molybdate using a UV-1800 spectrophotometer (Shimadzu, Japan) (Ren et al., 2017a). The calcium content of the excreta samples was analyzed with a Z-2000 flame atomic absorption spectrophotometer (Hitachi, Japan) (Erfanian et al., 2017). Percentage phosphorus and calcium concentrations of the excreta samples are presented on an air-dried basis, and 24-h total excretion of phosphorus and calcium were calculated accordingly.

#### Quantitative Real-Time PCR

The qPCR analysis was performed as previously described (Liu et al., 2018). Total RNA of the samples was extracted using TRIZOL reagent (Invitrogen, Carlsbad, CA, United States) according to the manufacturer’s specifications. The concentration and purity of the extracted RNA were determined using a NanoDrop 2000c spectrophotometer (Thermo Scientific, Wilmington, United States). Qualified RNA was subjected to cDNA synthesis using a Primer Script RT Reagent Kit (TaKaRa, Dalian, China). Then, mRNA expression levels of genes of interest were analyzed with a SYBR Premix Ex Taq kit (TaKaRa, Dalian, China) using an iCycler iQ5 real-time PCR machine (Bio-Rad, Hercules, CA): *FGF23* (calvaria and liver), *FGFR1-4* (calvaria, liver, intestine, and kidney), *klotho* (calvaria, liver, intestine, and kidney), *SLC34A1* (kidney; the gene coding for NPt2a), and *SLC34A2* (intestine; the gene coding for NPt2b). The sequences (Table 2) of primers used in quantitative real-time PCR analysis were designed with the Primer3 program based on gene sequences obtained from the National Center for Biotechnology Information. All reactions were run in triplicate. Relative mRNA expression was calculated to β-actin using the 2^–ΔΔCt^ method.

**TABLE 2:** Primer sequences for quantitative real-time PCR (experiment 2).

Genes	Accession No.	Sequences (5′–3′)
*FGF23*	XM_425663.4	F: ATGCTGCTTGTGCTCTGTATC
		R: ACTGTAAATGGTTTGGTGAGG
*FGFR1*	XM_015297362.2	F: CTTCTCCGTCAACGTCTCAG
		R: GTTCGGCTTGGTGTTATCC
*FGFR2*	XM_015288594.2	F: AGAACATCGTATTGGCGGCT
		R: GATCGCTCGACAACATCGAGA
*FGFR3*	XM_015285884.2	F: GGACCTGATGGGACACCCTA
		R: AGCTCCTCTGCTGGTTTTGG
*FGFR4*	XM_015293864.2	F: GCTCCCAGAAGAAGAGCTGG
		R: GTGCTGGACTTTCCCGATGA
*Klotho*	XM_417105.6	F: ACCCGTCAATCCTGTTGG
		R: TCAGCGTAGTCGTGGAAGAG
*SLC34A1*	XM_015293846.2	F: CCAAACTGCACGGCTTCT
		R: TGGGAGGTCAGTGTTGATGA
*SLC34A2*	NM_204474.2	F: GCCTGGTAAAGGTTTGGTGC
		R: TGCCGGCATTTAGTGATATATTCTG
β*-actin*	XM_029084520.1	F: ATTGTCCACGCAAATGCTTC
		R: AAATAAAGCCATGCCAACTCGTC

**FGF23*, fibroblast growth factor 23; *FGFR*, fibroblast growth factor receptor; *SLC34A1*, the gene coding for type IIa sodium-phosphate co-transporters (NPt2a); *SLC34A2*, the gene coding for type IIb sodium-phosphate co-transporters (NPt2b).*

#### Western Blot

Western blot analysis was performed as previously described (Liu et al., 2018). The protein components of the samples were extracted, and the protein concentrations were determined with a BCA protein assay kit (catalog no. WB003, Hat Biotechnology, Xi’an, China). The protein components were electrophoresed in 8% SDS-PAGE and transferred electrophoretically to PVDF Western blotting membranes (catalog no. 03010040001, Roche Diagnostics, Mannheim, Germany). The membranes were blocked and incubated with the primary antibody (diluted to 1:1000) for 1 h at room temperature and then overnight at 4°C. After washing, the secondary antibody (HRP-conjugated, diluted to 1:1000) was applied for 1.5 h. Then, the membranes were washed, probed, and autoradiographed with a Chemiluminescence gel imaging system (DNR, Micro Chemi, Israel) using a Super Signal West Pico Trial Kit (catalog no. 34580; Pierce, IL, United States). Rabbit antibodies to NPt2a (catalog no. A6742) and NPt2b (catalog no. A9460) were purchased from ABclonal Technology (Wuhan, China). Mouse antibody to β-actin was purchased from CWBio. Co., Ltd. (Beijing, China; catalog no. CW0096). The secondary antibodies were purchased from Bioss Biotechnology Co., Ltd. (Beijing, China; goat anti-mouse IgG, catalog no. bs-0296G-HRP), and Wuhan Diyi Biotechnology Co., Ltd. (Hubei, China; goat anti-rabbit IgG, catalog no. DY60202). The blot density (measured with Image J software 1.8.0) was normalized to β-actin.

### Statistical Analysis

Data were analyzed by either one-way ANOVA (experiment 1) or two-sided independent student’s *t*-test (experiment 2) using SPSS version 23.0 (IBM Corp., Chicago, IL, United States). In experiment 1, each replicate (12 birds) was considered as the statistical unit. In experiment 2, the individual laying hen was considered as the statistical unit. Results are presented as means and standard error of the mean. Statistical significance was considered at *P* < 0.05 and trends at *P* < 0.1. According to our previous data (Ren et al., 2017c), seven laying hens per treatment would be sufficient to reflect the influences of dietary phosphorus levels on serum phosphorus concentrations (two means, 1.32 compared to 2.11 mmol/L, with two-sided significance of *P* < 0.05, with a pooled standard error of 0.50). As expected, in experiment 2, a statistical power of > 0.80 and a two-sided significance of *P* < 0.001 were obtained on serum phosphate concentrations by sampling seven laying hens from each experimental diet.

## Results

### Experiment 1

As shown in Tables 3, 4, in experiment 1, dietary non-phytate phosphorus levels (0.12, 0.17, 0.22, 0.27, 0.32, 0.37, and 0.42%) had no effects on laying rate (*P* = 0.618), egg weight (*P* = 0.178), daily feed intake (*P* = 0.346), feed-to-egg ratio (*P* = 0.335), unqualified egg rate (*P* = 0.508), eggshell thickness (*P* = 0.598), eggshell strength (*P* = 0.953), or egg shape index (*P* = 0.157) in Hy-Line Brown laying hens during a 15-week feeding trial.

**TABLE 3:** Effect of dietary non-phytate phosphorus levels on laying performance in Hy-Line Brown layers (experiment 1)*.

Dietary non-phytate phosphorus (%)	Laying rate (%)	Egg weight (g)	Daily feed intake (g)	Feed-to-egg ratio (kg:kg)	Unqualified egg rate (%)
0.12	95.0	62.8	127.2	2.14	0.31
0.17	91.9	65.0	130.8	2.19	1.07
0.22	91.9	63.4	133.3	2.31	0.59
0.27	95.1	62.9	132.4	2.22	0.82
0.32	94.7	63.8	135.6	2.24	1.18
0.37	90.1	62.7	129.1	2.28	0.73
0.42	93.5	63.4	131.9	2.24	0.35
SEM	0.8	0.2	1.0	0.02	0.13
*P*-value*	0.618	0.178	0.346	0.335	0.508

*All of the data were subjected to linear and quadratic regression analyses, but no significance was recorded (*P* > 0.05). Unqualified egg rate = (total number of dirty, broken, softshell, and malformed eggs)/(total egg number).

**TABLE 4:** Effect of dietary non-phytate phosphorus levels on egg quality in Hy-Line Brown layers (experiment 1)*.

Dietary non-phytate phosphorus (%)	Eggshell thickness (mm)	Eggshell strength (g of force)	Egg shape index (cm:cm)
0.12	0.40	3934	1.31
0.17	0.41	3970	1.33
0.22	0.40	3972	1.31
0.27	0.40	4211	1.29
0.32	0.39	4018	1.30
0.37	0.39	3989	1.32
0.42	0.39	4146	1.32
SEM	0.01	73	0.01
*P*-value	0.598	0.953	0.157

*All of the data were subjected to linear and quadratic regression analyses, but no significance was recorded (*P* > 0.05).

### Experiment 2

#### Laying Performance and Egg Quality

As shown in Table 5, in experiment 2, decreasing dietary non-phytate phosphorus levels from 0.32% (regular phosphorus) to 0.14% (phosphorus restriction) had no effects on laying performance measured as laying rate (*P* = 0.300), egg weight (*P* = 0.746), daily feed intake (*P* = 0.861), and feed-to-egg ratio (*P* = 0.920) in Hy-Line Brown laying hens. Similarly, dietary non-phytate phosphorus restriction had no effects on egg quality parameters including shell thickness (*P* = 0.745), shell index (*P* = 0.484), shell strength (*P* = 0.340), albumen height (*P* = 0.839), yolk pigmentation (*P* = 0.669), Haugh units (*P* = 0.634), and specific gravity (*P* = 0.945). No adverse events were noticed during the study period.

**TABLE 5:** Effects of dietary phosphorus restriction on laying performance and egg quality in Hy-Line Brown layers (experiment 2)*.

Items	Phosphorus restriction	Regular phosphorus	*P*-value
**Laying performance**			
Laying rate (%)	98.0 ± 1.0	95.9 ± 1.6	0.300
Egg weight (g)	58.2 ± 1.3	58.9 ± 1.7	0.746
Daily feed intake (g)	116.5 ± 2.5	117.7 ± 6.8	0.861
Feed-to-egg ratio (kg:kg)	2.02 ± 0.04	2.01 ± 0.09	0.920
**Egg quality**			
Shell thickness (mm)	0.37 ± 0.02	0.37 ± 0.01	0.745
Shell index (% of whole egg)	11.3 ± 0.4	11.0 ± 0.3	0.484
Shell strength (g of force)	4255 ± 194	4663 ± 378	0.340
Albumen height (mm)	6.84 ± 0.61	7.00 ± 0.39	0.839
Yolk pigmentation	6.93 ± 0.19	6.80 ± 0.23	0.669
Haugh units	81.5 ± 3.5	83.6 ± 2.1	0.634
Specific gravity	1.090 ± 0.002	1.091 ± 0.002	0.945

*Phosphorus restriction = 0.14% dietary non-phytate phosphorus; regular phosphorus = 0.32% dietary non-phytate phosphorus.

#### Serum Analysis

As shown in Table 6, serum levels of phosphorus and FGF23 were decreased by 40.3% (*P* < 0.001) and 41.7% (*P* = 0.011), respectively, when dietary non-phytate phosphorus levels were decreased from 0.32 to 0.14%. Dietary non-phytate phosphorus restriction had no effects on serum levels of calcium (*P* = 0.762), AKP (*P* = 0.650), 1,25(OH)_2_D_3_ (*P* = 0.234), and PTH (*P* = 0.260) in Hy-Line Brown laying hens.

**TABLE 6:** Effects of dietary phosphorus restriction on serum phosphorus, calcium, and hormones in Hy-Line Brown layers (experiment 2)*.

Items	Phosphorus restriction	Regular phosphorus	*P*-value
Phosphorus (mmol/L)	0.89 ± 0.04^b^	1.49 ± 0.09^a^	<0.001
Calcium (mmol/L)	1.56 ± 0.04	1.58 ± 0.04	0.762
Alkaline phosphatase (King unit/100 mL)	23.42 ± 6.01	27.46 ± 6.23	0.650
FGF23 (pg/mL)	101.5 ± 13.4^b^	174.0 ± 20.2^a^	0.011
1,25(OH)_2_D_3_ (ng/mL)	69.6 ± 8.75	57.8 ± 3.35	0.234
PTH (pg/mL)	112.2 ± 11.3	97.7 ± 5.0	0.260

FGF23, fibroblast growth factor 23; 1,25(OH)_2_D_3_, 1,25-dihydroxy-cholecalciferol; PTH, parathyroid hormone. ^*a*,*b*^Means within a row with unlike superscript letters were significantly different (*P* < 0.05). *Phosphorus restriction = 0.14% dietary non-phytate phosphorus; regular phosphorus = 0.32% dietary non-phytate phosphorus.

#### Phosphorus and Calcium Excretion

**FIGURE 1:**
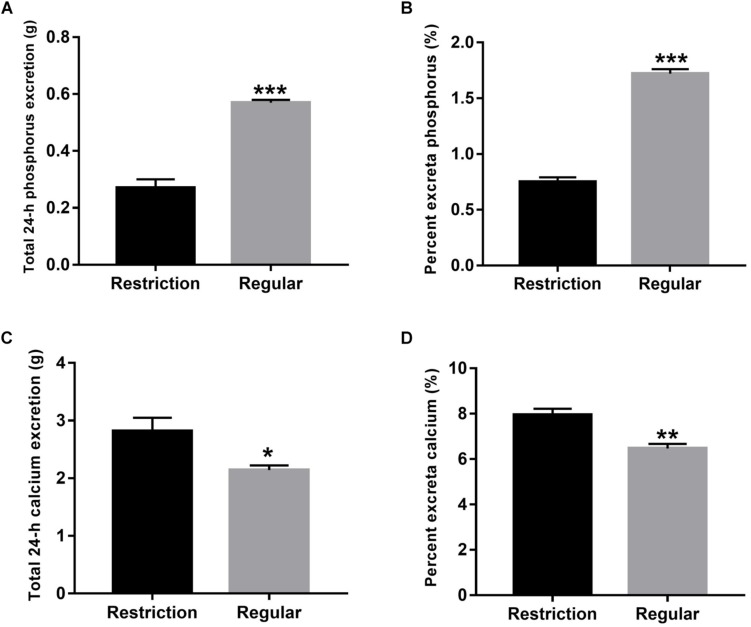
Effects of dietary non-phytate phosphorus levels (restriction, 0.14%; regular, 0.32%) on the excretion of phosphorus [**(A)** total 24-h phosphorus excretion and **(B)** percentage phosphorus in excreta] and calcium [**(C)** total 24-h calcium excretion and **(D)** percentage calcium in excreta] in laying hens. Values are of 7 hens/treatment. *P < 0.05; **P < 0.01; ***P < 0.001. Experiment 2.

As shown in Figure 1, percentage phosphorus in excreta was decreased by 56.4% (decreased from 1.72 to 0.75%, *P* < 0.001) and 24-h total phosphorus excretion was decreased by 52.6% (decreased from 0.57 to 0.27 g, *P* < 0.001) when dietary non-phytate phosphorus levels were decreased from 0.32 to 0.14% in Hy-Line Brown laying hens. Interestingly, percent calcium in excreta was increased by 22.9% (increased from 6.47 to 7.95%, *P* = 0.001) and 24-h total calcium excretion was increased by 31.8% (increased from 2.14 to 2.82 g, *P* = 0.024) when dietary non-phytate phosphorus levels were decreased from 0.32 to 0.14%.

#### mRNA Expressions of *FGF23*, *FGFR*, and *Klotho*

**FIGURE 2:**
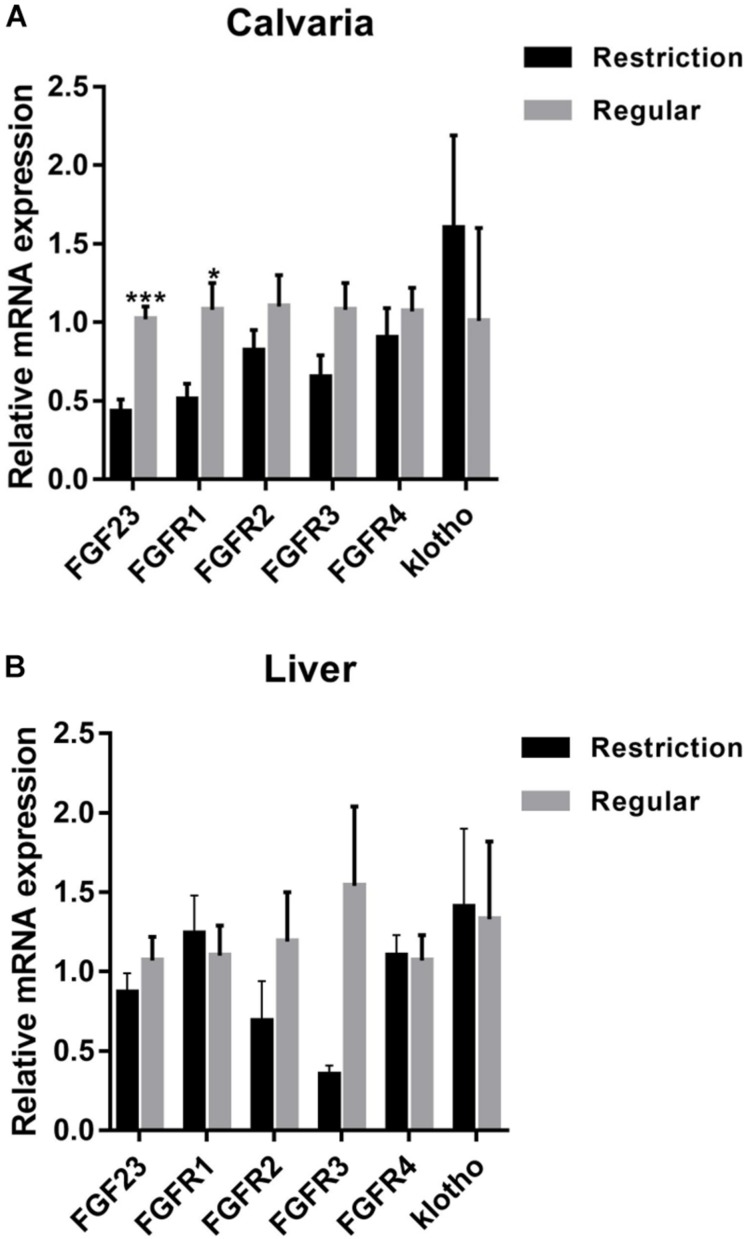
Effects of dietary non-phytate phosphorus levels (restriction, 0.14%; regular, 0.32%) on mRNA expressions of fibroblast growth factor 23 (*FGF23*) and its receptors in laying-hen calvaria **(A)** and liver **(B)**. Values are of 7 hens/treatment. *FGFR*, fibroblast growth factor receptor. *P < 0.05; ***P < 0.001. Experiment 2.

As shown in Figure 2, calvaria expressions of *FGF23* and *FGFR1* were decreased by 57.8% (*P* < 0.001) and 52.8% (*P* = 0.012), respectively, when dietary non-phytate phosphorus was decreased from 0.32 to 0.14%. Dietary restriction had no effects on calvaria expressions of *FGFR2* (*P* = 0.255), *FGFR3* (*P* = 0.075), *FGFR4* (*P* = 0.521), and *klotho* (*P* = 0.498), or on liver expressions of *FGF23* (*P* = 0.339), *FGFR1* (*P* = 0.661), *FGFR2* (*P* = 0.241), *FGFR3* (*P* = 0.055), *FGFR4* (*P* = 0.900), and *klotho* (*P* = 0.905).

**FIGURE 3:**
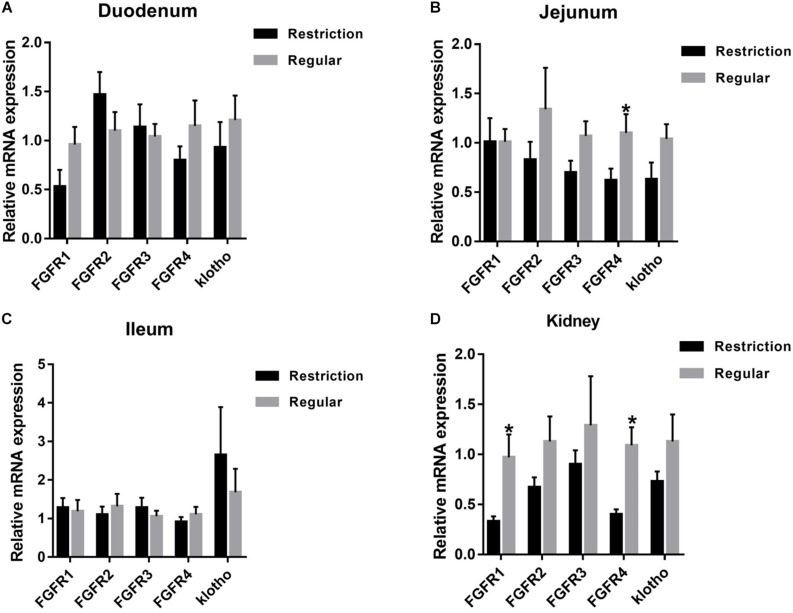
Effects of dietary non-phytate phosphorus levels (restriction, 0.14%; regular, 0.32%) on intestine **(A)** duodenum, **(B)** jejunum, **(C)** ileum, and kidney **(D)** mRNA expressions of fibroblast growth factor 23 (FGF23) receptors in laying hens. Values are of 7 hens/treatment. *FGFR*, fibroblast growth factor receptor. *P < 0.05. Experiment 2.

As shown in Figure 3, jejunum expressions of *FGFR4* were decreased by 43.6% (*P* = 0.047) and kidney expressions of *FGFR1* and *FGFR4* were decreased by 66.0% (*P* = 0.040) and 63.3% (*P* = 0.012), respectively, when dietary non-phytate phosphorus was decreased from 0.32 to 0.14%. Dietary restriction had no effects on jejunum expressions of *FGFR2* (*P* = 0.293), *FGFR3* (*P* = 0.076), and *klotho* (*P* = 0.100) or on kidney expressions of *FGFR2* (*P* = 0.123), *FGFR3* (*P* = 0.462), and *klotho* (*P* = 0.191). Dietary non-phytate phosphorus levels had no effects on duodenum and ileum expressions of *FGFR1* (duodenum *P* = 0.079, ileum *P* = 0.828), *FGFR2* (duodenum *P* = 0.240, ileum *P* = 0.565), *FGFR3* (duodenum *P* = 0.731, ileum *P* = 0.448), *FGFR4* (duodenum *P* = 0.259, ileum *P* = 0.394), and *klotho* (duodenum *P* = 0.450, ileum *P* = 0.500).

#### Intestine mRNA and Protein Expressions of SLC34A2 (NPt2b)

**FIGURE 4:**
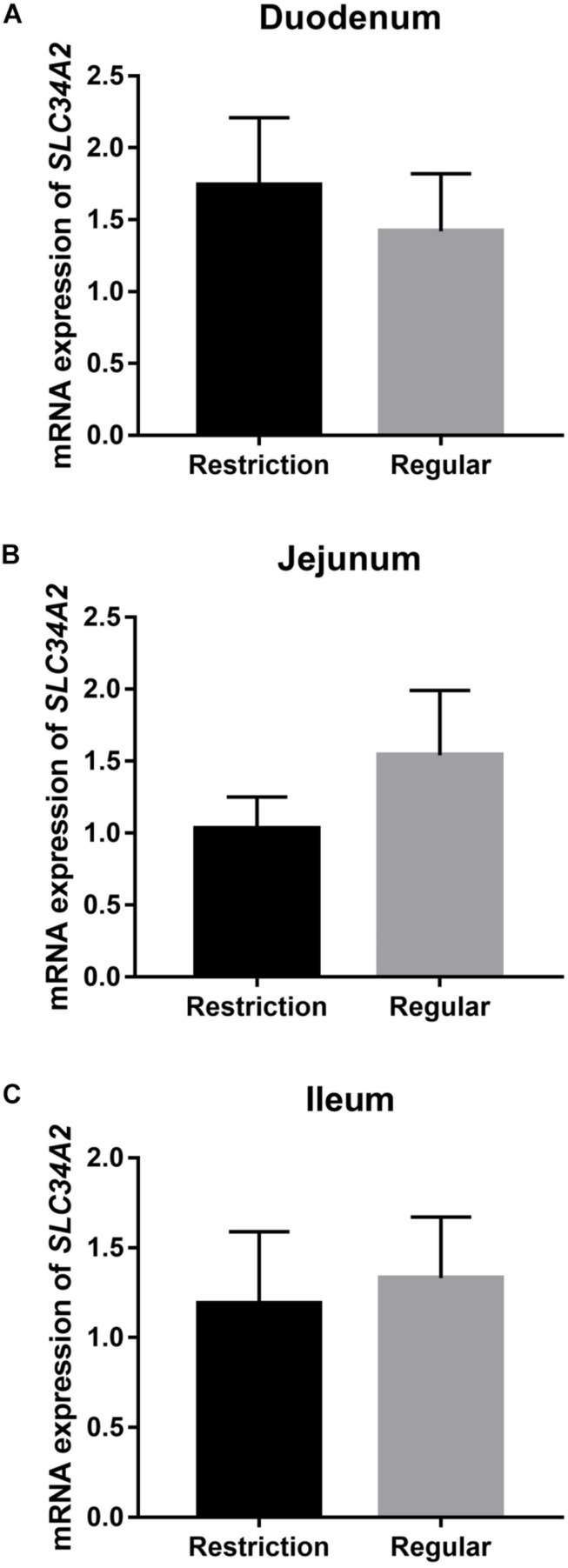
Effects of dietary non-phytate phosphorus levels (restriction, 0.14%; regular, 0.32%) on intestine **(A)** duodenum, **(B)** jejunum, and **(C)** ileum mRNA expressions of *SLC34A2* in laying hens. *SLC34A2* is the gene coding for type 2b sodium-phosphorus co-transporter (NPt2b). Values are of 7 hens/treatment. Experiment 2.

**FIGURE 5:**
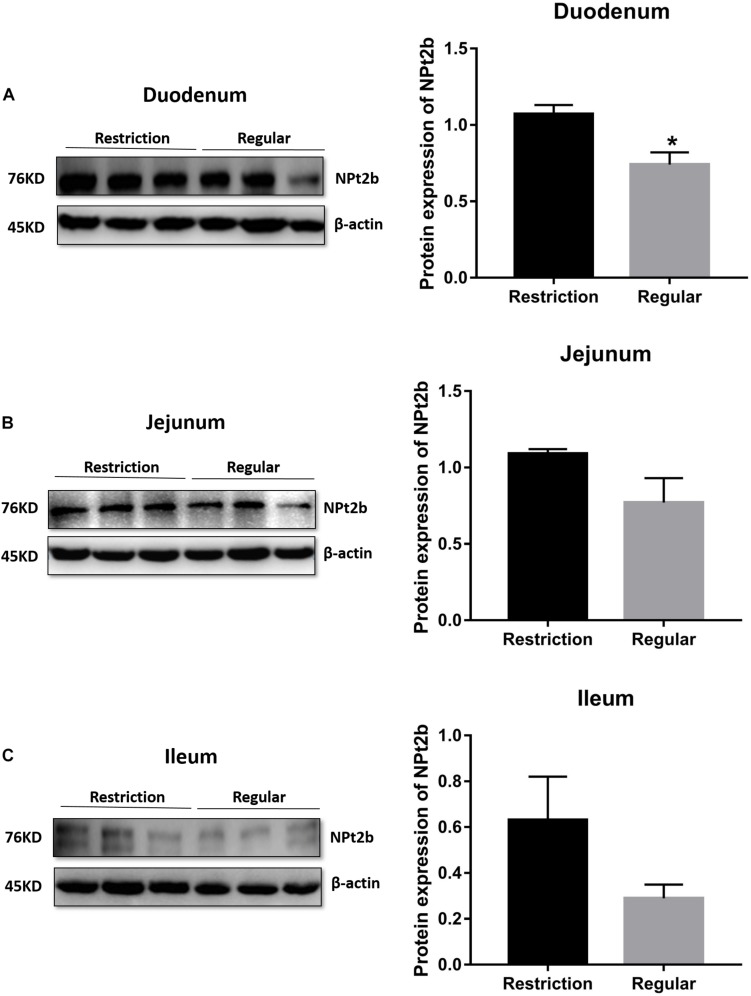
Effects of dietary non-phytate phosphorus levels (restriction, 0.14%; regular, 0.32%) on intestine **(A**) duodenum, **(B)** jejunum, and **(C)** ileum protein expressions of type 2b sodium-phosphorus co-transporter (NPt2b) in laying hens. Values are of 3 hens/treatment. *P < 0.05. Experiment 2.

Decreasing dietary non-phytate phosphorus levels from 0.32 to 0.14% had no effects on duodenum (*P* = 0.617, Figure 4), jejunum (*P* = 0.330, Figure 4), and ileum (*P* = 0.781, Figure 4) mRNA expressions of *SLC34A2* or on jejunum (*P* = 0.123, Figure 5B) and ileum (*P* = 0.174, Figure 5C) protein expressions of NPt2b but did significantly increase protein expression of NPt2b in duodenum (*P* = 0.032, Figure 5A) in Hy-Line Brown laying hens.

#### Kidney mRNA and Protein Expressions of SLC34A1 (NPt2a)

**FIGURE 6:**
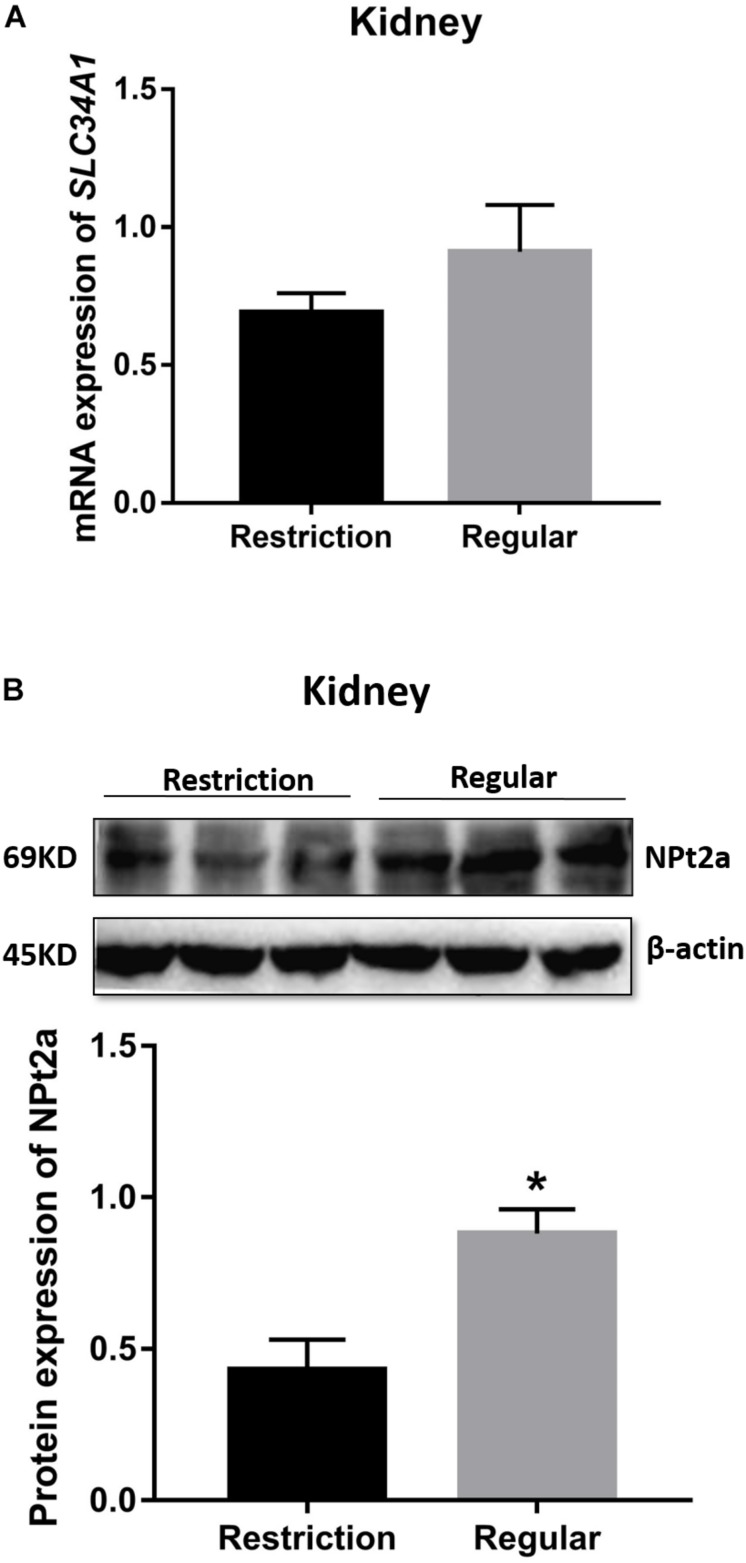
Effects of dietary non-phytate phosphorus levels (restriction, 0.14%; regular, 0.32%) on kidney expressions *SLC34A1* mRNA **(A)** and NPt2a protein **(B)** in laying hens. *SLC34A1* is the gene coding for type 2a sodium-phosphorus co-transporter (NPt2a). Values are of 3 hens/treatment. *P < 0.05. Experiment 2.

As shown in Figure 6, decreasing dietary non-phytate phosphorus levels from 0.32 to 0.14% had no effects on kidney mRNA expression of *SLC34A1* (*P* = 0.266) but significantly decreased kidney protein expression of NPt2a (*P* = 0.025) in Hy-Line Brown laying hens.

## Discussion

Phosphorus restriction had no effects on laying performance and egg quality in the current study. Similar results were reported when comparing 0.15% to 0.45% (Jing et al., 2018), suggesting that the current speculations regarding dietary phosphorus requirements will need to be re-evaluated in laying hens. The National Research Council (National Research Council [NRC], 1994), Chinese Feeding Standard of Chicken (NY/T 33-2004), and Hy-Line Brown Layer Management Guide (2016) recommend dietary non-phytate phosphorus concentrations for laying hens of 0.25, 0.32, and 0.45%, respectively. With 6.6 billion laying hens raised worldwide (Food and Agriculture Organization of the United Nations [FAO], 2013), decreasing dietary non-phytate phosphorus from 0.25–0.45% to 0.14% would save 0.29–0.82 million tons of inorganic phosphorus, a strategic material for many countries (Vaccari et al., 2019), every single year. Some have estimated that the domestic animal manure represents >15 million tons of phosphorus excretion globally per year (Cordell et al., 2009). In this study, when decreasing dietary non-phytate phosphorus from 0.32% to 0.14%, the percentage phosphorus in excreta and 24-h total phosphorus excretion of laying hens were decreased by 56.4% and 52.6%, respectively, indicating the significant importance of a phosphorus-reducing diet formulating strategy in environmental protection (Martin et al., 2018; Mekonnen and Hoekstra, 2018). Indeed, it has been repeatedly mentioned in the literature that reducing non-phytate phosphorus levels in laying-hen diets would save phosphorus without negatively affecting productivity (Jing et al., 2018) and health status (Ahmadi and Rodehutscord, 2012; Jing et al., 2018). However, since the physiological mechanisms of response to low-phosphorus diets have not been clearly illustrated, the poultry industry continues applying a large safety margin for laying hens. Below, we demonstrate that the existence of FGF23 grants avian species the ability to live on diets with a wide range of phosphorus concentrations and allows them to adapt well to low-phosphorus diets (e.g., 0.14% non-phytate phosphorus for egg-laying hens).

The serum phosphorus level is sensitive to dietary phosphorus consumption (Perwad et al., 2005). In humans, the normal physiological concentration range of serum samples has been well established (e.g., 0.85 to 1.15 mmol/L, adults in China) and has long been used to evaluate body phosphorus nutrition and metabolism status (Kalantar-Zadeh et al., 2018). However, while no information is available regarding the normal physiological concentration range of chickens, poultry nutritionists keep using serum phosphorus levels to evaluate the phosphorus requirements of laying hens (Jing et al., 2018). Basically, decreased levels of serum phosphorus, regardless of whether or not it is within the normal physiological range of chicken, have been considered as an indicator of impaired phosphorus nutrition status (Tabeekh et al., 2016). In the current experiment 2, phosphorus restriction decreased serum phosphorus by 40.3% (from 1.49% to 0.89%) but, interestingly, had no negative effects on laying performance and egg quality. Also, we found that decreasing dietary non-phytate phosphorus levels from 0.42% to 0.14% had no effects on laying performance, egg quality and tibia quality in laying hens from 29 to 40 weeks of age (Cheng et al., unpublished results) and from 40 to 55 weeks of age (experiment 2). These results indicate that laying hens could adapt well to diets that support a serum phosphorus level of 0.89%. The important point to be noted is that it is possible that a lower phosphorus concentration in serum may cause decreasing productivity if the feeding trial continues for a long time (e.g., the whole egg-laying period). Thus, the minimal non-phytate phosphorus needs of laying hens will need to be carefully validated in field trials. Of particular interest is the finding that decreased serum phosphorus levels were accompanied by decreases in serum FGF23 levels (decreased by 41.7%), demonstrating a well-accepted fact that the body enhances FGF23 production in order to induce phosphorus excretion in case of dietary phosphorus load (Pool and Wolf, 2017). In animals (e.g., wild birds) where dietary phosphorus is in overload, FGF-23 has critical functions in reducing the serum phosphorus concentration and alleviating phosphorus toxicity (Pool and Wolf, 2017). However, in the modern laying-hen industry, dietary phosphorus levels can be precisely controlled to assure both animal and environmental health, and the phosphorus-reducing function of FGF23 can actually interfere with the poultry nutritionists’ goals of maximizing body phosphorus retention. In this sense, feeding laying hens high non-phytate phosphorus diets (e.g., 0.32%) will not further improve egg-producing efficiency but may increase the burden of phosphorus excretion. In the literature, increased levels of serum phosphorus and FGF23 levels have been linked to unexpected outcomes such as CKD (Musgrove and Wolf, 2019), hyperparathyroidism (Chou et al., 2019), and cardiovascular events (Schwantes-An et al., 2019) in humans.

Bone appears to be a principal source of FGF23 in humans and mice (Faul, 2018). Interestingly, in laying hens, the highest *FGF23* mRNA expression level was detected in liver when compared to other tissues such as bone, brain, spleen, intestine, heart, and kidney (Wang et al., 2018). For chicken bone tissues, calvaria expresses the highest levels of *FGF23* mRNA, followed with femur, tibia, and medullary bone (Wang et al., 2018). In the current study, increasing dietary non-phytate phosphorus levels increased *FGF23* mRNA expression in calvaria but had no effect on liver *FGF23* mRNA expression in laying hens. These results indicate that bone-sourced FGF23 plays primary roles in responding to changes in phosphorus nutrition status in laying hens. What remains unclear is the effect of dietary non-phytate phosphorus levels on *FGF23* mRNA expression in other bone tissues. Also, the physiological importance of liver-sourced FGF23 in laying hens and the reason why liver expresses the highest levels of *FGF23* mRNA but is not sensitive to dietary phosphorus changes will need to be investigated in further studies.

Fibroblast growth factor 23 has been shown to directly target the kidney to stimulate urine phosphorus excretion (Gattineni et al., 2009). The presence of FGF23 receptors FGFR1, FGFR3, FGFR4, and klotho has been previously confirmed in the renal proximal tubule (Gattineni et al., 2009; Hu et al., 2010). In this study, kidney mRNA expressions of *FGFR1* and *FGFR4* were decreased when decreasing dietary non-phytate phosphorus from 0.32% to 0.14%. While the mechanisms by which dietary phosphorus consumption regulates kidney *FGFR* expressions remain to be investigated, along with the decreased *FGF23* mRNA expression in the calvaria and the decreased levels of serum FGF23, these results suggest that body FGF23 production was significantly suppressed in 0.14% non-phytate phosphorus-fed laying hens. In the literature, decreases in circulating phosphorus and FGF23 levels have been linked to increases in kidney expression of NPt2a (Gattineni et al., 2009), which is responsible for phosphorus resorption in the renal proximal tubule (Werner et al., 1998; Gagnon et al., 2019). In the current study, the transcriptional level of kidney *NPt2a* mRNA was not affected by dietary treatments; however, kidney NPt2a protein expression was decreased by 51% in laying hens fed 0.14% non-phytate phosphorus when compared to those fed 0.32% non-phytate phosphorus. To our best knowledge, there is little/no information on the effects of dietary non-phytate phosphorus levels on renal NPt2a protein production in laying hens. In Bourgeois et al. (2013), after mice were switched to a low-phosphorus diet, the renal NPt2a protein abundance was decreased by 26% at 2 h but increased by 43% at 4 h. In Thomas et al. (2017), after rats were orally gavaged with phosphate, the renal NPt2a protein abundance was increased by 42% at 40 minutes but decreased by 51% at 4 h. These observations suggest that, in the early period of low-phosphorus stimulation, renal NPt2a protein abundance might be decreased. In laying hens, the serum phosphorus status changes dynamically during the daily egg-laying cycle (Ren et al., 2019). In the current study, laying hens were sampled shortly after oviposition, a time point with the lowest serum phosphorus levels in the day (Ren et al., 2019). So, it is possible that, in addition to dietary non-phytate phosphorus levels, the daily egg-laying cycle also interfered in renal NPt2a protein production, thus leading to the current observations on NPt2a protein abundance in laying hens.

Fibroblast growth factor 23 inhibits renal production of 1,25(OH)_2_D_3_ and subsequently decreases phosphorus absorption by suppressing intestinal expression of NPT2b (Shimada et al., 2004; Sabbagh et al., 2009). In this study, while statistical significance was not detected (*P* = 0.234), serum 1,25(OH)_2_D_3_ levels were increased by 20% in laying hens fed with 0.14% non-phytate phosphorus. As expected, duodenum, jejunum, and ileum expressions of NPt2b protein were increased by 45%, 42%, and 117%, respectively. These results indicated that the laying hens’ adaption to a low-phosphorus diet is a result of enhanced intestinal phosphorus absorption efficiency but not renal resorption efficiency. Interestingly, when increasing dietary phosphorus levels from 0.14% to 0.32%, the increased serum FGF23 levels were accompanied by inhibited intestinal NPt2b expression at the post-transcriptional level but not at the transcriptional level. Similarly, it is reported that intestinal NPt2b activity is controlled by regulating protein abundance and membrane trafficking but not by modifying mRNA expression, suggesting post-transcriptional and protein distribution control (Hu et al., 2010, 2018). In retrospect, the intestine has not been identified as a direct target organ for FGF23 (Edmonston and Wolf, 2019). So, the decreased mRNA expression of jejunum *FGFR4* in 0.14% non-phytate phosphorus-fed laying hens will need to be explained in future studies. Decreasing dietary non-phytate phosphorus levels decreased phosphorus excretion but increased calcium excretion in laying hens, suggesting that dietary calcium levels may need to be reconsidered when formulating environmentally friendly low-phosphorus diets for laying hens.

## Conclusion

In conclusion, we demonstrate that decreasing dietary non-phytate phosphorus levels (experiment 1, decreased from 0.42% to 0.12%; experiment 2, decreased from 0.32% to 0.14%) had no negative effects on laying performance and egg quality but significantly decreased phosphorus excretion (decreased by 52.6% in experiment 2) in Hy-Line Brown laying hens. The laying hens adjusted to a low-phosphorus diet by decreasing serum FGF23 concentration and subsequently increasing intestinal NPt2b protein production. These results underscore the importance of controlling dietary phosphorus concentrations or omitting overdosing of the nutrient in feed manufacture to preserve non-renewable phosphorus and prevent environmental pollution by phosphorus from the animal excreta.

## Data Availability Statement

All datasets generated for this study are included in the article/supplementary material.

## Ethics Statement

The animal study was reviewed and approved by the College of Animal Science and Technology Animal Care and Use Committee at the Northwest A&F University.

## Author Contributions

ZR and XJY developed the research idea and designed the project. ZR and JY carried out the study, analyzed the data, and wrote the manuscript. QH, XL, CP, YL, and XZ contributed to animal care and sample analysis. XY helped with data analysis and manuscript revision.

## Conflict of Interest

The authors declare that the research was conducted in the absence of any commercial or financial relationships that could be construed as a potential conflict of interest.

## Abbreviations

1,25(OH)_2_D_3_: 1,25-dihydroxyvitamin D_3_
AKP: alkaline phosphatase
CKD: chronic kidney disease
FGF23: Fibroblast growth factor 23
FGFR: fibroblast growth factor receptor
NPt2: type 2a sodium-phosphorus co-transporter
NRC: National Research Council
PTH: parathyroid hormone.

## References

[B1] Ahmadi H. Rodehutscord M. (2012). A meta-analysis of responses to dietary nonphytate phosphorus and phytase in laying hens. *Poult. Sci.* 91 2072–2078. 10.3382/ps.2012-02193 22802206

[B2] Bai X. Y. Miao D. S. Goltzman D. Karaplis A. C. (2003). The autosomal dominant hypophosphatemic rickets R176Q mutation in fibroblast growth factor 23 resists proteolytic cleavage and enhances in vivo biological potency. *J. Biol. Chem.* 278 9843–9849. 10.1074/jbc.m210490200 12519781

[B3] Bougouin A. Appuhamy J. Kebreab E. Dijkstra J. Kwakkel R. France J. (2014). Effects of phytase supplementation on phosphorus retention in broilers and layers: a meta-analysis. *Poult. Sci.* 93 1981–1992. 10.3382/ps.2013-03820 24902701

[B4] Bourgeois S. Capuano P. Stange G. Mühlemann R. Murer H. Biber J. (2013). The phosphate transporter NaPi-IIa determines the rapid renal adaptation to dietary phosphate intake in mouse irrespective of persistently high FGF23 levels. *Pflüg Arch* 465 1557–1572. 10.1007/s00424-013-1298-9 23708836

[B5] Chou F. F. Chen J. B. Huang S. C. Chan Y. C. Chi S. Y. Chen W. T. (2019). Changes in serum FGF23 and Klotho levels and calcification scores of the abdominal aorta after parathyroidectomy for secondary hyperparathyroidism. *Am. J. Surg.* 218 609–612. 10.1016/j.amjsurg.2018.12.026 30594298

[B6] Cordell D. Drangert J. O. White S. (2009). The story of phosphorus: global food security and food for thought. *Global Environ. Chang.* 19 292–305. 10.1016/j.gloenvcha.2008.10.009

[B7] Edmonston D. Wolf M. (2019). FGF23 at the crossroads of phosphate, iron economy and erythropoiesis. *Nat. Rev. Nephrol.* 16 7–19. 10.1038/s41581-019-0189-5 31519999

[B8] Erfanian A. Rasti B. Manap Y. (2017). Comparing the calcium bioavailability from two types of nano-sized enriched milk using in-vivo assay. *Food Chem.* 214 606–613. 10.1016/j.foodchem.2016.07.116 27507516

[B9] Food and Agriculture Organization of the United Nations [FAO] (2013). *Statistical yearbook: world food and agriculture* Rome: FAO, 140–145.

[B10] Faul C. (2018). FGF23 effects on the heart-levels, time, source, and context matter. *Kidney Int.* 94 7–11. 10.1016/j.kint.2018.03.024 29933856

[B11] Fink G. Alcamo J. Flörke M. Reder K. (2018). Phosphorus loadings to the world’s largest lakes: sources and trends. *Global Biogeochem. Cycles* 32 617–634. 10.1002/2017gb005858

[B12] Gagnon K. B. Barati M. T. Kitterman K. Clark B. Lederer E. D. (2019). Forward trafficking of NPT2a in the renal proximal tubule is inhibited by increased association of intracellular proteins identified by proteomic analysis. *FASEB J.* 33 817–824.

[B13] Gattineni J. Bates C. Twombley K. Dwarakanath V. Robinson M. L. Goetz R. (2009). FGF23 decreases renal NaPi-2a and NaPi-2c expression and induces hypophosphatemia in vivo predominantly via FGF receptor 1. *Am. J. Physiol. Renal* 297 282–291. 10.1152/ajprenal.90742.2008 19515808 PMC2724258

[B14] Goyette J. O. Bennett E. Maranger R. (2018). Low buffering capacity and slow recovery of anthropogenic phosphorus pollution in watersheds. *Nat. Geosci.* 11 921–925. 10.1038/s41561-018-0238-x

[B15] Hu M. C. Shi M. Moe O. W. (2018). Role of αKlotho and FGF23 in regulation of type II Na-dependent phosphate co-transporters. *Pflug Arch* 471 99–108. 10.1007/s00424-018-2238-5 30506274 PMC6324980

[B16] Hu M. C. Shi M. Zhang J. Pastor J. Nakatani T. Lanske B. (2010). Klotho: a novel phosphaturic substance acting as an autocrine enzyme in the renal proximal tubule. *FASEB J.* 24 3438–3450. 10.1096/fj.10-154765 20466874 PMC2923354

[B17] Hy-Line Brown Layer Management Guide (2016). *Des Moines*. Iowa, IA: Hy-Line International.

[B18] Jing M. Zhao S. Rogiewicz A. Slominski B. A. House J. D. (2018). Assessment of the minimal available phosphorus needs of laying hens: implications for phosphorus management strategies. *Poult. Sci.* 97 2400–2410. 10.3382/ps/pey057 29617962

[B19] Kalantar-Zadeh K. Parameswaran V. Ficociello L. H. Anderson L. Ofsthun N. J. Kwoh C. (2018). Real-world scenario improvements in serum phosphorus levels and pill burden in peritoneal dialysis patients treated with sucroferric oxyhydroxide. *Am. J. Nephrol.* 47 153–161. 10.1159/000487856 29514139 PMC5906196

[B20] Kuro-o M. (2019). The Klotho proteins in health and disease. *Nat. Rev. Nephrol.* 15 27–44. 10.1038/s41581-018-0078-3 30455427

[B21] Levi M. Gratton E. Forster I. C. Hernando N. Wagner C. A. Biber J. (2019). Mechanisms of phosphate transport. *Nat. Rev. Nephrol.* 15 482–500. 10.1038/s41581-019-0159-y 31168066

[B22] Liu Y. Shen J. Yang X. Sun Q. Yang X. (2018). Folic acid reduced triglycerides deposition in primary chicken hepatocytes. *J. Agric. Food Chem.* 66 13162–13172. 10.1021/acs.jafc.8b05193 30484310

[B23] Martin K. L. Emanuel R. E. Vose J. M. (2018). Terra incognita: the unknown risks to environmental quality posed by the spatial distribution and abundance of concentrated animal feeding operations. *Sci. Total Environ.* 642 887–893. 10.1016/j.scitotenv.2018.06.072 29929140

[B24] Mekonnen M. M. Hoekstra A. Y. (2018). Global anthropogenic phosphorus loads to freshwater and associated grey water footprints and water pollution levels: a high-resolution global study. *Water Resour. Res.* 54 345–358. 10.1002/2017wr020448

[B25] Michigami T. Kawai M. Yamazaki M. Ozono K. (2018). Phosphate as a signaling molecule and its sensing mechanism. *Physiol. Rev.* 98 2317–2348. 10.1152/physrev.00022.2017 30109818

[B26] Musgrove J. Wolf M. (2019). Regulation and effects of FGF23 in chronic kidney disease. *Annu. Rev. Physiol.* [Epub ahead of print]. 31743079 10.1146/annurev-physiol-021119-034650

[B27] National Research Council [NRC] (1994). *Nutrient Requirements of Poultry*, 9th Edn. Washington, DC: National Academic Press, 19–34.

[B28] Perwad F. Azam N. Zhang M. Y. H. Yamashita T. Tenenhouse H. S. Portale A. A. (2005). Dietary and serum phosphorus regulate fibroblast growth factor 23 expression and 1,25-dihydroxyvitamin D metabolism in mice. *Endocrinology* 146 5358–5364. 10.1210/en.2005-0777 16123154

[B29] Pool L. R. Wolf M. (2017). FGF23 and nutritional metabolism. *Annu. Rev. Nutr.* 37 247–268. 10.1146/annurev-nutr-071816-064620 28715994

[B30] Portale A. A. Booth B. E. Halloran B. P. Morris R. (1984). Effect of dietary phosphorus on circulating concentrations of 1, 25-dihydroxyvitamin D and immunoreactive parathyroid hormone in children with moderate renal insufficiency. *J. Clin. Invest.* 73 1580–1589. 10.1172/jci111365 6547151 PMC437069

[B31] Ren Z. Bütz D. E. Sand J. M. Cook M. E. (2017a). Maternally-derived anti-fibroblast growth factor 23 antibody as new tool to reduce phosphorus requirement of chicks. *Poult. Sci.* 96 878–885. 10.3382/ps/pew314 27647928

[B32] Ren Z. Bütz D. E. Wahhab A. N. Piepenburg A. J. Cook M. E. (2017b). Additive effects of fibroblast growth factor 23 neutralization and dietary phytase on chick calcium and phosphorus metabolism. *Poult. Sci.* 96 1167–1173. 10.3382/ps/pew339 27665015

[B33] Ren Z. Z. Ebrahimi M. Butz D. E. Sand J. M. Zhang K. Y. Cook M. E. (2017c). Antibody to fibroblast growth factor 23-peptide reduces excreta phosphorus of laying hens. *Poult. Sci.* 96 127–134. 10.3382/ps/pew189 27287381

[B34] Ren Z. Jiang S. Zeng Q. Ding X. Bai S. Wang J. (2016). Effect of dietary canthaxanthin and 25-hydroxycholecalciferol supplementation on the performance of duck breeders under two different vitamin regimens. *J. Anim. Sci. Biotechnol.* 7:2. 10.1186/s40104-016-0062-3 26807215 PMC4724121

[B35] Ren Z. Z. Piepenburg A. J. Bütz D. E. Claus J. R. Cook M. E. (2018). Vaccine to fibroblast growth factor 23 peptides increases eggshell strength. *Poult. Sci.* 97 882–889. 10.3382/ps/pex373 29272435

[B36] Ren Z. Z. Sun W. Q. Liu Y. L. Li Z. P. Han D. Cheng X. (2019). Dynamics of serum phosphorus, calcium, and hormones during egg laying cycle in Hy-line brown laying hens. *Poult. Sci.* 98 2193–2200. 10.3382/ps/pey572 30608558

[B37] Sabbagh Y. O’Brien S. P. Song W. P. Boulanger J. H. Stockmann A. Arbeeny C. (2009). Intestinal npt2b plays a major role in phosphate absorption and homeostasis. *J. Am. Soc. Nephrol.* 20 2348–2358. 10.1681/ASN.2009050559 19729436 PMC2799172

[B38] Schwantes-An T. H. Liu S. Stedman M. Decker B. S. Wetherill L. Edenberg H. J. (2019). Fibroblast growth factor 23 genotype and cardiovascular disease in patients undergoing hemodialysis. *Am. J. Nephrol.* 49 125–132. 10.1159/000496060 30669147 PMC6473180

[B39] Shimada T. Hasegawa H. Yamazaki Y. Muto T. Hino R. Takeuchi Y. (2004). FGF-23 is a potent regulator of vitamin D metabolism and phosphate homeostasis. *J. Bone. Miner. Res.* 19 429–435. 10.1359/jbmr.0301264 15040831

[B40] Tabeekh A. Mudhar A. Abbas R. J. (2016). The effect of color light and stocking density on tibial measurements and levels of calcium and phosphorus in bone and serum of broilers and layers chickens. *In. J. Sci. Technol.* 143 1–7.

[B41] Thomas L. Bettoni C. Knöpfel T. Hernando N. Biber J. Wagner C. A. (2017). Acute adaption to oral or intravenous phosphate requires parathyroid hormone. *J. Am. Soc. Nephrol.* 28 903–914. 10.1681/ASN.2016010082 28246304 PMC5328153

[B42] Urakawa I. Yamazaki Y. Shimada T. Iijima K. Hasegawa H. Okawa K. (2006). Klotho converts canonical FGF receptor into a specific receptor for FGF23. *Nature* 444 770–774. 10.1038/nature05315 17086194

[B43] Vaccari D. A. Powers S. M. Liu X. (2019). Demand-driven model for global phosphate rock suggests paths for phosphorus sustainability. *Environ. Sci. Technol.* 53 10417–10425. 10.1021/acs.est.9b02464 31393113

[B44] Wang R. M. Zhao J. P. Wang X. J. Jiao H. C. Wu J. M. Lin H. (2018). Fibroblast growth factor 23 mRNA expression profile in chickens and its response to dietary phosphorus. *Poult. Sci.* 97 2258–2266. 10.3382/ps/pey092 29688456

[B45] Werner A. Dehmelt L. Nalbant P. (1998). Na+-dependent phosphate cotransporters: the NaPi protein families. *J. Exp. Biol.* 201 3135–3142. 9808829 10.1242/jeb.201.23.3135

[B46] White K. E. Evans W. E. O’Riordan J. L. Speer M. C. Econs M. J. Lorenz-Depiereux B. (2000). Autosomal dominant hypophosphataemic rickets is associated with mutations in FGF23. *Nat. Genet.* 26 345–348. 10.1038/81664 11062477

